# Supplementary material for: Stable representation of sounds in the posterior striatum during flexible auditory decisions
Source: Nat Commun. 2018 Apr 18;9:1534. doi: 10.1038/s41467-018-03994-3 (PMC5906458; doi:10.1038/s41467-018-03994-3)
Supplement: Supplementary file 1 — Supplementary Information [file 41467_2018_3994_MOESM1_ESM.pdf]

Stable representation of sounds in the posterior striatum during flexible auditory decisions

Guo et al.

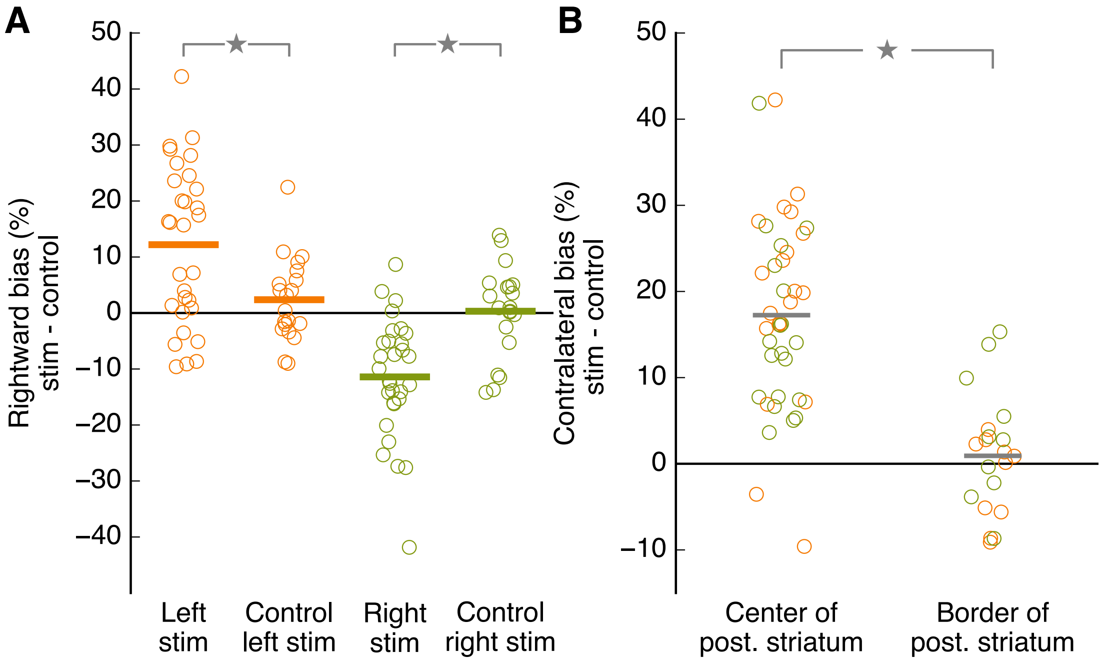

Supplementary Figure 1. Laser stimulation did not produce behavioral bias in wild-type mice. Related to Figure 2. (A) Change in the percentage of rightward choices during laser stimulation for each hemisphere in *Drd1a::ChR2* mice and wild-type control mice. Each dot represents one session (*Drd1a::ChR2*: N=3 mice, 10 sessions each hemisphere per mouse; wild-type control: N=2 mice, 10 sessions each hemisphere per mouse). Horizontal bars represent averages across all sessions for all mice. Stimulation produced significantly different biases in the *Drd1a::ChR2* mice versus the control mice in each hemisphere ($p = 0.028$ left, $p < 0.001$ right, Wilcoxon rank-sum test). (B) Comparison of contralateral bias resulting from activation of different sites on the medial-lateral axis of posterior striatum in *Drd1a::ChR2* mice. Stimulation of sites at the center of the striatum produced significantly higher bias than sites near the border between striatum and cortex ($p < 0.001$, Wilcoxon rank-sum test).

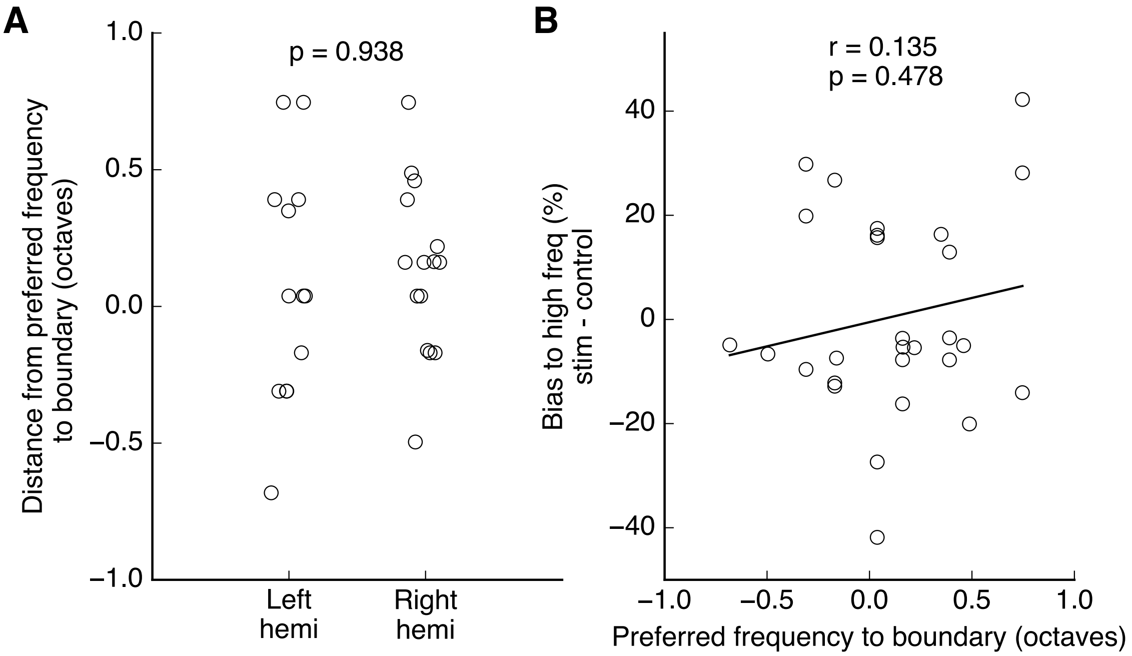

Supplementary Figure 2. Behavioral bias did not correlate with frequency preference of optical stimulation sites. Related to Figure 2. (A) Preferred sound stimuli (relative to categorization boundary) at optogenetic stimulation sites in each hemisphere of *Drd1a::ChR2* mice. Each dot represents the multiunit activity recorded from a stimulus-selective site. Frequency tuning was not significantly different between hemispheres ($p = 0.938$, Wilcoxon rank-sum test). (B) There was no significant correlation between choice bias and frequency tuning at a stimulated site ($r = 0.135$, $p = 0.478$, Spearman correlation test).

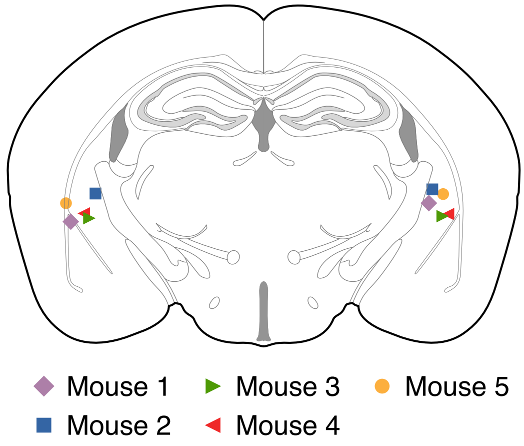

Supplementary Figure 3. Center of muscimol injection in the posterior striatum for each mouse tested. Related to Figure 3. After the last behavioral session, a fluorescent dye was injected and the center of the injection estimated from fixed brain sections.

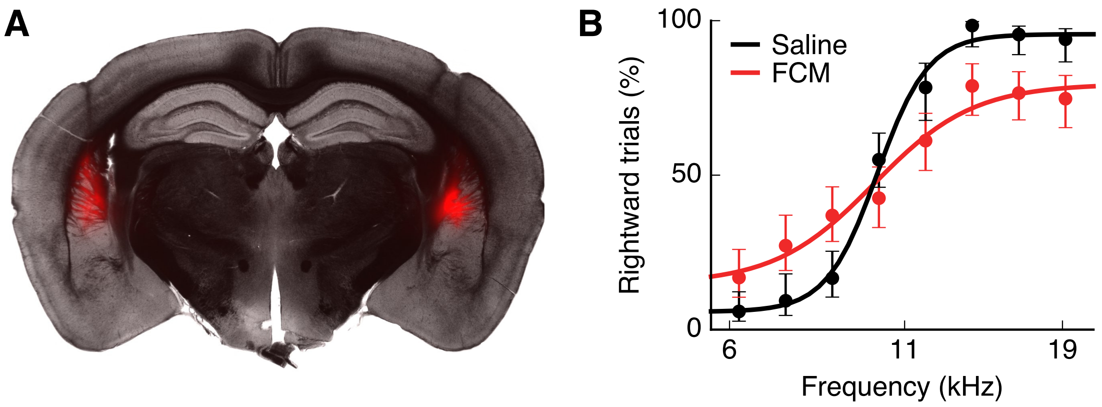

Supplementary Figure 4. Inactivation of posterior striatum using fluorescent muscimol impaired task performance. Related to Figure 3. (A) Coronal section showing injection site and spread of Muscimol-BODIPY TMR-X Conjugate (Fluorescent Conjugated Muscimol, FCM) in the posterior striatum. The rostrocaudal spread was approximately 0.8 mm centered at the injection site. (B) Average psychometric performance of the mouse in (A) after injection of FCM (red, 1 session) or saline control (black, 1 session). Error bars indicate 95% confidence intervals.

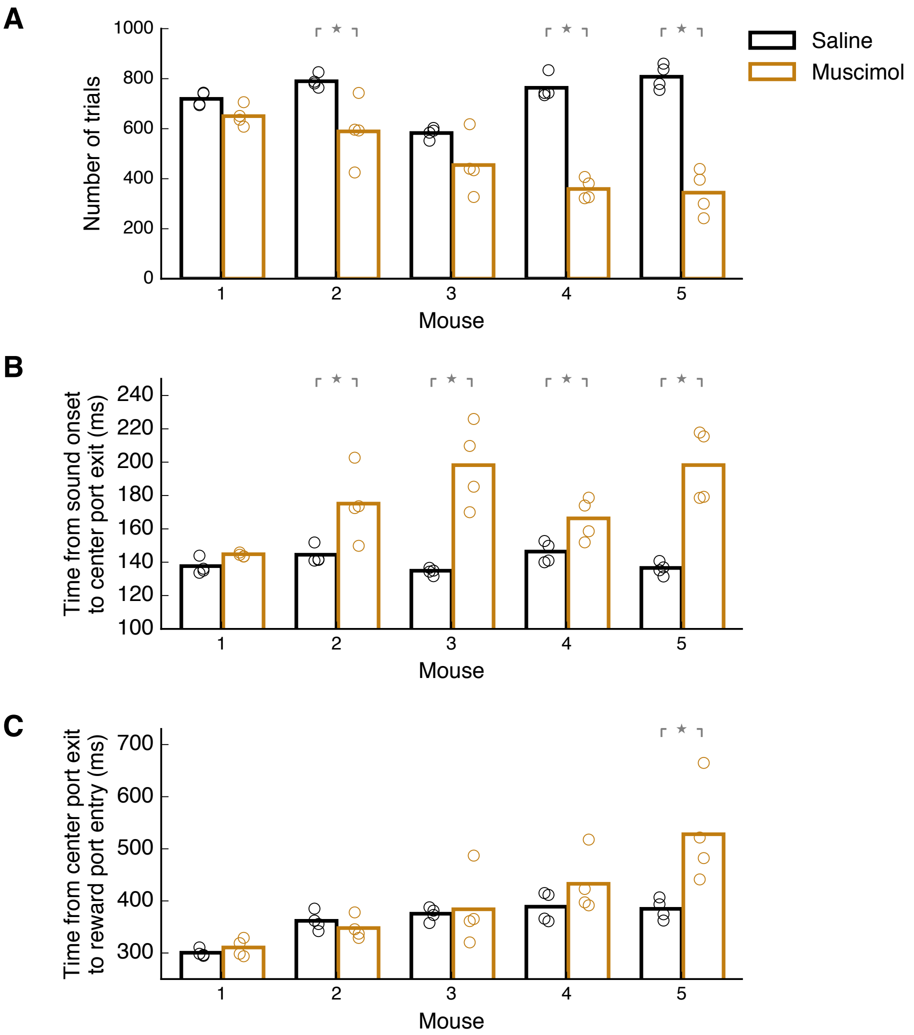

Supplementary Figure 5. Effect of muscimol inactivation of posterior striatum on movement during the sound discrimination task. Related to Figure 3. (A) Number of trials performed in 1 hour on each saline session (black) and each muscimol session (brown) for each mouse. Bars indicate average across sessions for each mouse. Muscimol inactivation significantly reduced the number of trials performed by 3 out of 5 mice ($p = 0.021$, Wilcoxon rank-sum test), but did not preclude animals from performing hundreds of trials in each session. (B) Average withdrawal time from center port after sound onset on each session. Muscimol inactivation led to slower withdrawals from the center port for four out of five mice ($p < 0.05$, Wilcoxon rank-sum test) compared to saline sessions. (C) Average movement time from center port to side reward ports on each session. A change in movement speed was observed in only one out of five mice ($p < 0.05$, Wilcoxon rank-sum test).

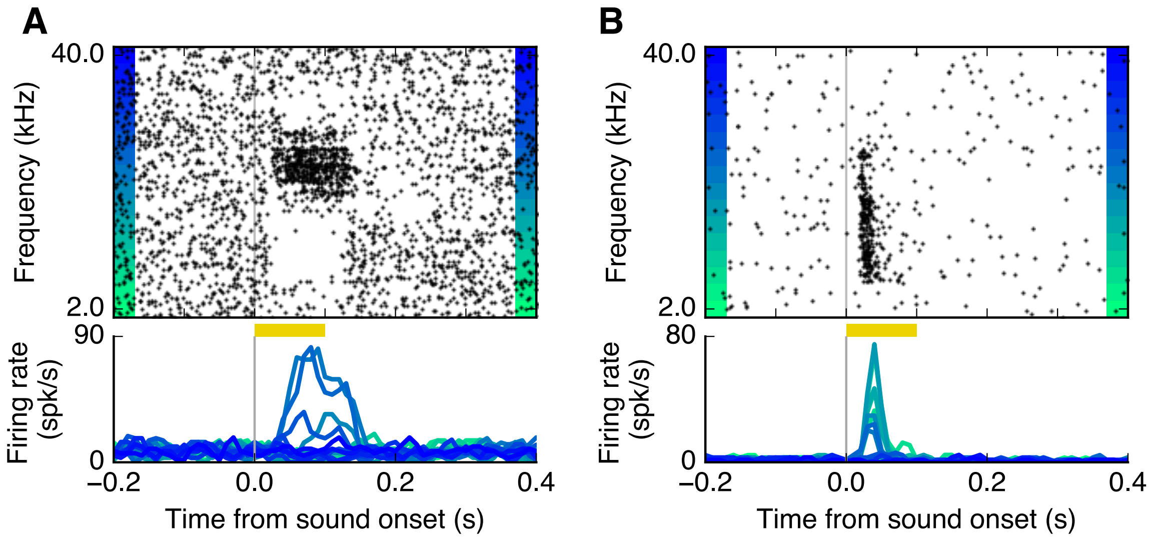

Supplementary Figure 6. Posterior striatal neurons displayed frequency-selective sound-evoked responses outside the sound discrimination task. Related to Figure 4. (A,B) Examples of sound responses from two different posterior striatal neurons to different frequencies of sound outside the sound discrimination task. Yellow bar indicates the duration of the sound (100 ms). Cells showed clear frequency selectivity.

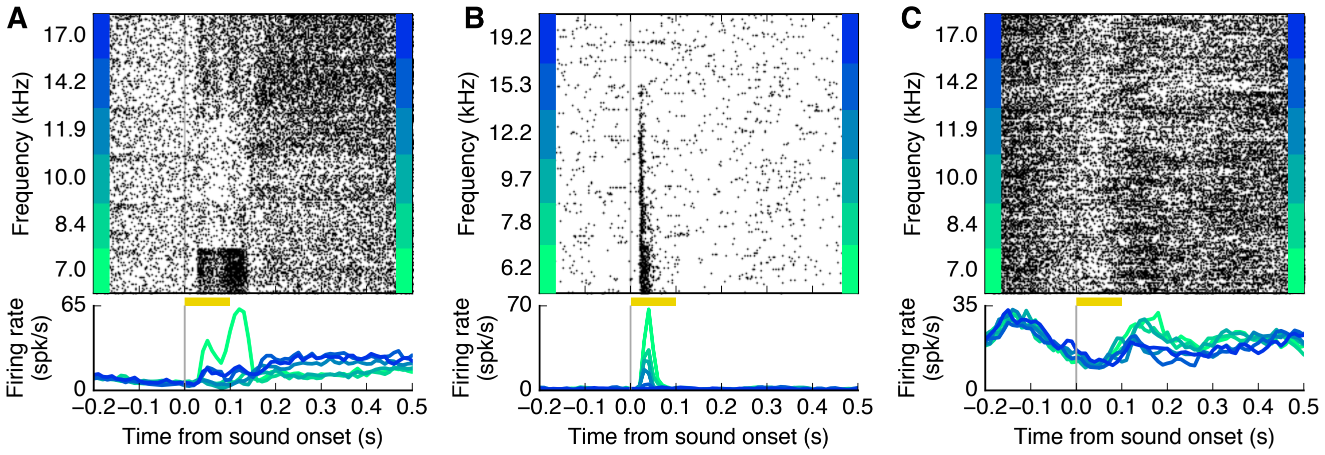

Supplementary Figure 7. Neuronal encoding of sound stimuli and movement direction in the posterior striatum. Related to Figure 4 and Figure 5. (A) Example cell that was both selective to sound frequency and movement direction. The plot includes trials in which the mouse went to the left port for the 3 lower frequencies and to the right port for the 3 higher frequencies. (B) Example cell that responded to sound stimuli, but was not sensitive to movement direction. (C) Example cell that did not respond to sound but was selectively active when animal was moving to one side port versus the other.

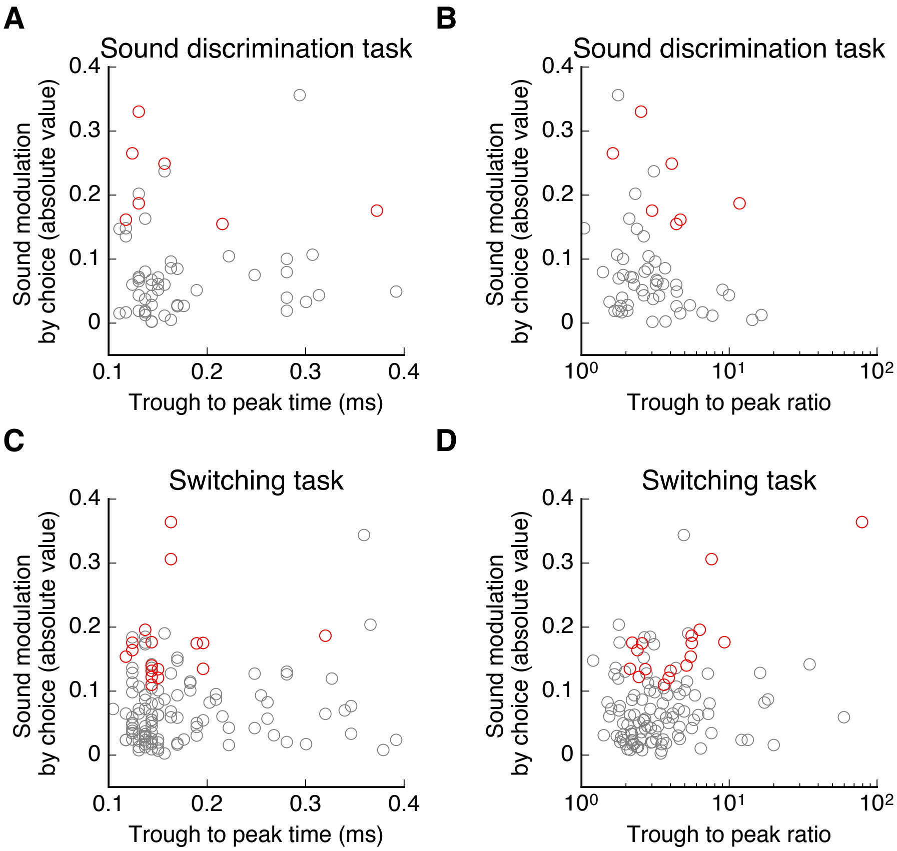

Supplementary Figure 8. Spike waveform parameters did not predict whether a cell's sound response was modulated by choice. Related to Figure 6 and Figure 7. (A) Spike trough-to-peak time was not correlated to the modulation index of sound by choice in the case of ambiguous stimuli (N = 67 cells from 5 mice, $r = -0.02$, $p = 0.8$, Spearman correlation test). (B) Modulation index of a cell was not correlated with its trough-to-peak ratio (N = 67 cells from 5 mice, $r = -0.2$, $p = 0.09$, Spearman correlation test). (C-D) Similarly, when mice were required to rapidly update stimulus-action association (switching task), sound response modulation index was not correlated with either trough-to-peak time ($r = 0.06$, $p = 0.5$, Spearman correlation test) or trough-to-peak ratio (N = 155 cells from 4 mice, $r = 0.17$, $p = 0.06$, Spearman correlation test).

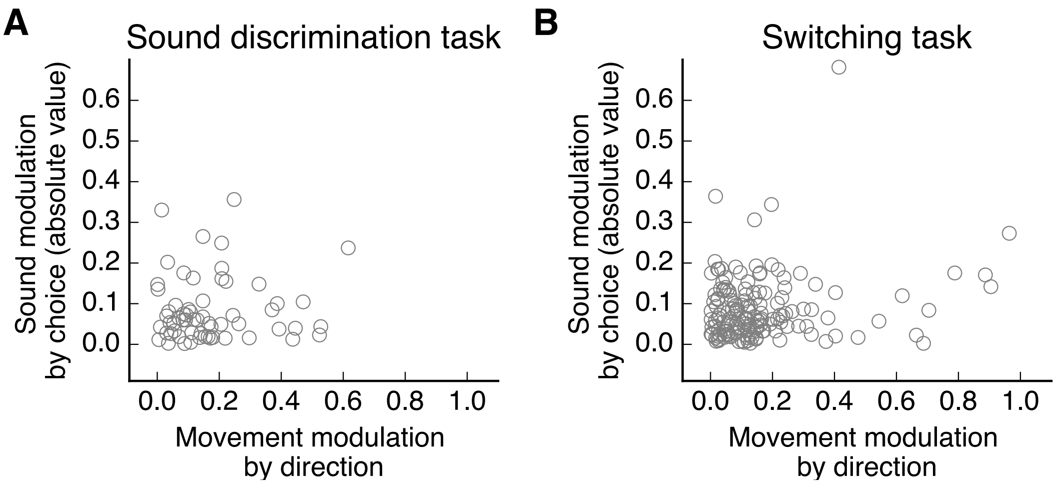

Supplementary Figure 9. Movement direction selectivity did not predict whether a cell's sound response was modulated by choice. Related to Figure 6 and Figure 7. (A) For cells that were responsive to sound stimuli near the categorization boundary, the magnitude of movement selectivity index did not correlate with that of the sound modulation index (N = 67 cells from 5 mice, $r = 0.02$, $p = 0.8$, Spearman correlation test). (B) For cells responsive to the stimulus that reversed associated in the switching task, the magnitude of movement selectivity index did not correlate with that of the sound modulation index (N = 155 cells from 4 mice, $r = 0.07$, $p = 0.4$, Spearman correlation test).

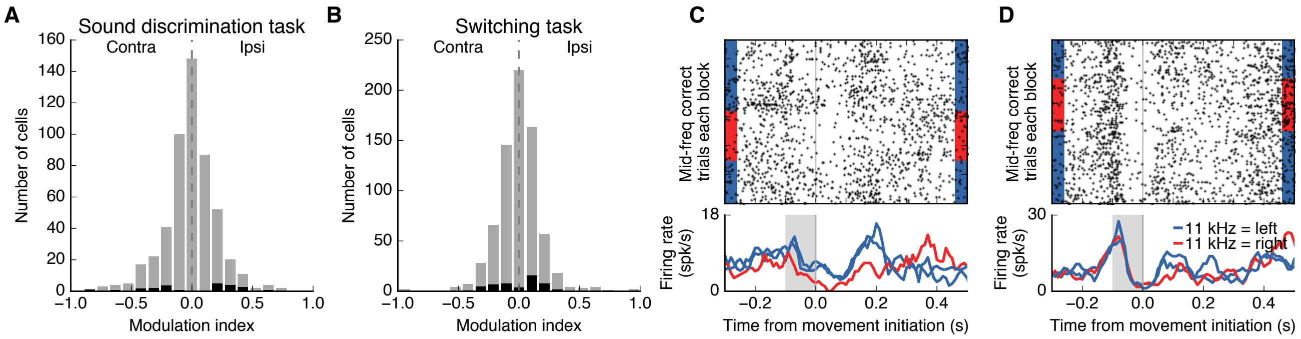

Supplementary Figure 10. Neuronal activity prior to movement initiation did not predict choice. Related to Figure 6 and Figure 7. (A) Influence of choice on neuronal activity before action initiation (100 ms period prior to the mouse exiting the center port) for ambiguous sound stimulus in the sound discrimination task (N = 520 cells from 5 mice). Less than 5% of neurons showed a significant change in activity before movement predictive of subsequent choice ($p < 0.05$, Wilcoxon rank-sum test), shown in black. (B) Influence of changing sound-action association for the stimulus of intermediate frequency on neuronal activity before action initiation (100 ms period prior to the mouse exiting the center port, N = 725 cells from 4 mice). Only 7% of neurons showed a significant change across sound-action contingencies ($p < 0.05$, Wilcoxon rank-sum test), shown in black. (C) Responses of one posterior striatal neuron aligned to movement start (0 on x-axis) in trials with the stimulus of intermediate frequency for three blocks of trials (switching task). Activity in the 0-100 msec window before movement initiation (shaded area) for this neuron changed systematically depending on the rewarded action associated with the stimulus. (D) Pre-movement activity of a different neuron showing no change across sound-action contingencies.

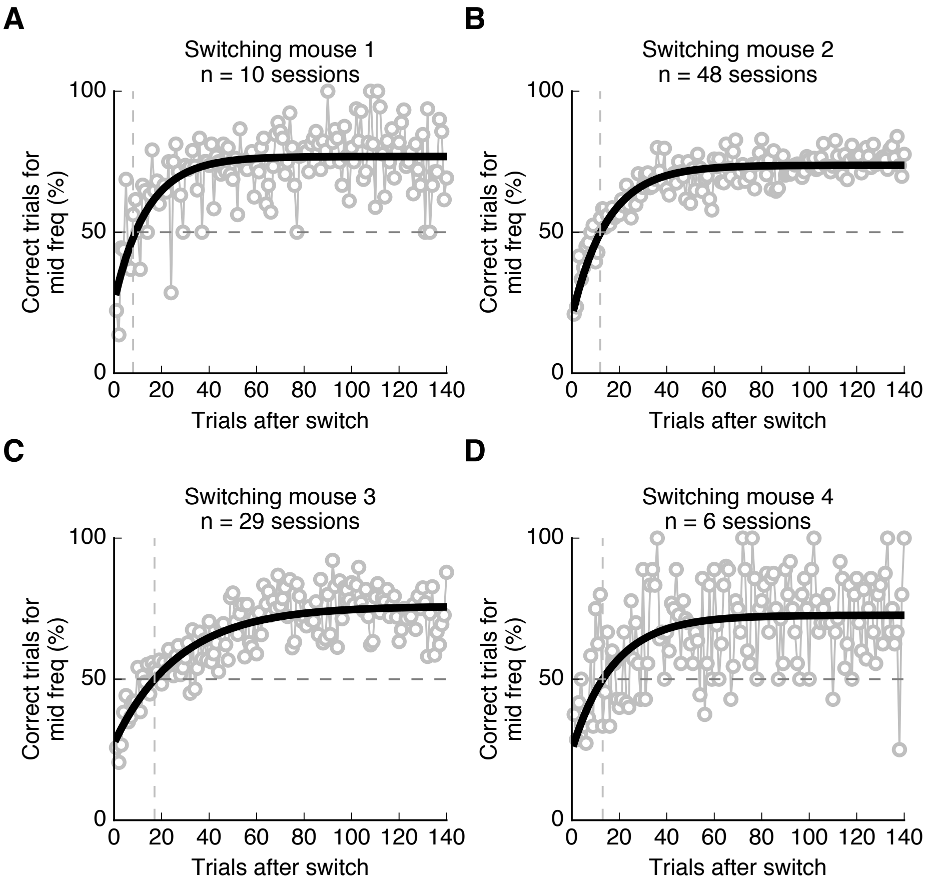

Supplementary Figure 11. Well-trained mice took less than 20 trials to switch after a contingency change. Related to Figure 7. Each panel shows performance of one mouse after the contingency switch. In gray is the average performance for each trial of the middle frequency across several sessions. In black is an exponential fit. The vertical dotted line indicates when performance crosses 50% accuracy level.
